# Rural and Urban Dwelling Across the Life-Course and Late-Life Cognitive Ability in Mexico

**DOI:** 10.1093/geroni/igaa057.1905

**Published:** 2020-12-16

**Authors:** Joseph Saenz

**Affiliations:** University of Southern California, Los Angeles, California, United States

## Abstract

BACKGROUND: Research has consistently suggested urban dwelling in late adulthood is associated with better cognitive ability. Whether early life rural/urban dwelling and its interaction with late-life rural/urban dwelling relate with late-life cognitive ability in the context of Mexico is not well understood. METHOD: Data comes from the 2003 Mexican Health and Aging Study. Early life rural/urban was assessed as respondents’ reports of growing up in an urban/rural area. Current rural/urban was assessed by locality size (greater/fewer than 100,000 residents). RESULTS: Both early life and current rural residence were associated with poorer cognitive ability independent of education, literacy, early life SES and health, income/wealth, healthcare access, health, and health behaviors. Compared to individuals who always lived in rural areas, rural to urban migration was associated with better cognitive ability. DISCUSSION: In addition to current rural/urban dwelling, researchers should consider where individuals lived in early life and migration across the life-course.

